# Is RANKL inhibition both anti-resorptive and anabolic in rheumatoid arthritis?

**DOI:** 10.1186/s13075-015-0861-5

**Published:** 2015-11-17

**Authors:** Natalie A. Sims, Evange Romas

**Affiliations:** St. Vincent’s Institute of Medical Research, 9 Princes Street, Fitzroy, VIC 3065 Australia; Department of Medicine at St. Vincent’s Hospital Melbourne, The University of Melbourne, 41 Victoria Pde, Fitzroy, VIC 3065 Australia; Department of Rheumatology, St. Vincent’s Hospital Melbourne, 41 Victoria Pde, Fitzroy, VIC 3065 Australia

## Abstract

A small peptide, OP3-4, blocks receptor activator of NF-κB from binding to its ligand, receptor activator of NF-κB ligand (RANKL), and was reported recently to inhibit bone resorption, promote bone formation and protect cartilage in a preclinical rheumatoid arthritis model. The latter effects may result from inhibition of RANKL reverse signalling in osteoblasts and chondrocytes. Whether other RANKL inhibitors, such as denosumab, share this action is not known, but OP3-4 at least has potential to provide anabolic treatment for both systemic and focal bone loss in inflammatory arthritis.

## Editorial

In a recent article in *Arthritis Research and Therapy*, Kato et al. [1] report anabolic action of a novel inhibitor of receptor activator of NF-κB ligand (RANKL) in a preclinical rheumatoid arthritis (RA) model.

Elevated osteoclast formation in RA occurs in two contexts: local osteoclastogenesis causing joint erosion and periarticular bone loss fuelled by tumour necrosis factor alpha (TNFα) and RANKL; and systemic bone resorption resulting in generalized osteoporosis [2].

To achieve low RA disease activity or remission, RA treatment must rapidly suppress inflammatory synovitis, initially with disease-modifying antirheumatic drugs (DMARDs) such as methotrexate and, if needed, followed by antibody-based biological agents, such as TNFα or interleukin (IL)-6 inhibitors (e.g. tocilizumab). The extent to which joint structure is protected from bone erosion with methotrexate correlates with synovitis suppression. In contrast, TNFα or IL-6 inhibitors abolish osteoclast-mediated bone erosion even with residual synovial inflammation, because IL-6 and TNFα stimulate osteoclast differentiation [2].

Osteoporosis in RA correlates with disease severity. Although bone loss may be prevented by treatment with methotrexate and TNFα inhibitors, bone antiresorptive therapy, specifically targeting osteoclasts, is often required to prevent fragility fractures [2]. Generally, weaker antiresorptives such as alendronate may preserve bone mineral density but do not prevent articular bone erosions. In contrast, zoledronate and RANKL inhibitors, such as denosumab, reduce osteoclast numbers, arresting both local erosion and systemic bone loss in preclinical models [3, 4] and in RA patients [5, 6]. These agents are not registered as DMARDs and denosumab has not generally been combined with biological DMARDs due to infection concerns. However, the hospitalized infection rate among RA patients receiving denosumab concurrently with biological DMARDs is no greater than in those receiving zoledronate [7].

Denosumab and zoledronate not only reduce bone resorption, but also inhibit serum bone formation markers in women with osteoporosis [8, 9]. This reflects a major function of osteoclasts beyond bone resorption: the production of ‘coupling factors’ and ‘osteotransmitters’ that promote bone formation on trabecular [10] and periosteal [11] surfaces, respectively. Increased bone mineral density observed during sustained osteoclast inhibition has therefore been thought to result not from increased bone formation, but from continued secondary mineralization in the absence of bone resorption [12].

The novel RANKL inhibitor used by Kato et al. [1] not only reduced bone resorption but also promoted bone formation and suppressed cartilage loss, suggesting a positive local effect on bone formation. This questions whether secondary mineralization is the only contributor to increased bone mineral density observed with RANKL inhibition.

The possibility that RANKL inhibition could promote bone formation was first identified when W9, a small molecule inhibitor of RANK-RANKL binding, not only impaired osteoclastogenesis but also promoted osteoblast differentiation in vitro, and stimulated cortical bone formation in vivo [13]. Follow-up studies in RANKL-deficient osteoblasts suggested that ‘outside-in’ or ‘reverse’ intracellular RANKL signalling within osteoblast precursors inhibits their differentiation [13]. Kato et al. [1] report that OP3-4, which also binds RANKL, not only inhibits bone resorption but increases bone formation in the collagen-induced arthritis model. This was particularly evident in the epiphysis, where local bone formation levels were low. OP3-4 also inhibited osteoblast differentiation in vitro [1]. Since hypertrophic chondrocytes express RANKL [14], OP3-4 may protect against cartilage destruction by inhibiting reverse RANKL signalling; preliminary data in a chondrocyte cell line are shown.

The precise mechanisms by which OP3-4 elicits an osteoblastic anabolic response via reverse RANKL signalling remain to be defined. It will also be important to determine whether OP3-4 promotes bone formation systemically, in specific locations (e.g. cortical or trabecular bone) or only in apposition to focal erosions in arthritis. From a clinical perspective, interaction of RANKL inhibition with anti-inflammatory approaches (including both synthetic small molecule and biological DMARDs) must be established.

Finally, a major question is whether the ability of OP3-4 and W9 to promote bone formation is shared with antibodies to RANKL such as denosumab. The current evidence suggests that this property is unique to the OP3-4 and W9 peptides. Recent histomorphometry in denosumab-treated cynomolgus monkeys showed that denosumab neither reduces bone modelling (bone formation on surfaces that have not been resorbed previously), nor stimulates bone formation [15].

Targeting RANKL to treat bone loss in inflammatory arthritis could provide more benefit than simply inhibiting resorption. Kato et al. highlight additional effects to promote bone formation and protect cartilage that deserve additional study.

## Abbreviations

DMARD: Disease-modifying antirheumatic drug
IL: Interleukin
RA: Rheumatoid arthritis
RANKL: Receptor activator of NF-κB ligand
TNFα: Tumour necrosis factor alpha
